# Examples of the Use of the ARAMIS 3D Measurement System for the Susceptibility to Deformation Tests for the Selected Mixtures of Coal Mining Wastes

**DOI:** 10.3390/s21134600

**Published:** 2021-07-05

**Authors:** Konrad Walotek, Joanna Bzówka, Adrian Ciołczyk

**Affiliations:** Department of Geotechnics and Roads, Faculty of Civil Engineering, Silesian University of Technology, Akademicka 5, 44-100 Gliwice, Poland; joanna.bzowka@polsl.pl (J.B.); adrian.ciolczyk@polsl.pl (A.C.)

**Keywords:** digital image correlation (DIC), ARAMIS 3D system, deformation measurement, strength test, coal mining waste, rubber waste

## Abstract

This paper presents the ARAMIS 3D system and examples of deformation susceptibility test results made on mixtures of coal mining waste and recycled tire rubber bound with the use of hydraulic binders. The ARAMIS 3D system is a measurement tool based on 3D scanning of the surface of the tested material. On the basis of the obtained 3D video image, the system allows for the continuous observation of the displacements occurring on the surface of the tested object during its load. This allows for a very detailed determination of the deformation distribution during the material loading. These types of measurement systems can be very useful, especially in the case of testing composite materials and testing materials under cyclic load conditions.

## 1. Introduction

Nowadays, researchers use modern systems such as digital image correlation (DIC) to carry out various types of measurement. The ARAMIS 3D system belongs to a group of methods that utilize the DIC system. The DIC system and its wide application was first introduced by Sutton et al. (2009) [1]. Pan (2018) [2] and many other researchers (for example [3,4,5,6,7,8,9,10,11,12]) have demonstrated its use as a non-contact image-based optical method for measuring full-field shape, displacement and deformation. DIC techniques first acquire digital images of an object at different loadings, different times or states using digital imaging devices including, among others, optical imaging, electronic imaging, and scanning probe imaging. Then, image analysis is performed with correlation-based matching (tracking or registration) algorithms and numerical differentiation approaches to quantitatively extract full-field displacement and strain responses of materials, components, structures or biological tissues. The 3D digital image correlation method is widely used for displacement measurements in laboratory conditions and for experimental applications in industries.

There are a huge number of publications dedicated to digital image correlation applications. Sutton et al. (2009) [1] have presented the history of the use of this method in various areas and a wide range of analyses have been carried out with the use of DIC. The following examples of DIC applications are known:

- In experimental mechanics for crack tip displacement field measurement, tip measurement of strain distribution of shape memory alloys, bridge deflection measurement [13,14,15];
- In the biomechanical field [4,16];
- In the geotechnical field, metal characterization, control tests [4,17,18];
- deformation measurements by DIC: correlation of a second-order displacement gradient [19];
- In biological tissues [20];
- For strain measurements in open-cell structures such as trabecular bone. A 3D DIC uses high-resolution computed tomography images for displacement measurements in the solid structure [21];
- The investigation of three types of the most commonly used sub-pixel displacement registration algorithms in terms of the registration accuracy and the computational efficiency using computer-simulated speckle images [22];
- Studying crack propagation in brittle materials such as ceramics [23];
- For studying subset size selection in DIC for speckle patterns [24];
- 2D DIC for in-plane displacement and strain measurement [25];
- Monitoring the crack growth process during a cyclic fatigue life [26];
- Analyzing the deformation mechanisms under transverse compression in a fi-ber-reinforced composite [27];
- In experiments including electron microscopes and stereo-microscopes, and large-scale application with a field of view of tens of meters, at low speed and at high- and ultra-high speed [28];
- In local and global approaches to DIC [29];
- For surface deformation measurements [2];
- In masonry wall measurement [30];
- For concrete beam measurement [31,32];
- In renewable energy sources’ installations testing [33].

An example of a DIC measurement tool is the ARAMIS de-vice. This paper presents the potential for measuring the deformation of the studied objects by means of DIC systems, using the ARAMIS 3D measurement system as an example.

Compression or tensile testing in a uniaxial state of stress is a basic test used for determining the strength parameters of materials used in construction. The method for performing these tests is precisely described in the standards related to the characteristics of the tested materials [34,35,36,37]; despite its improvement over the years, the method had not changed significantly. As a result of the strength test in the uniaxial stress state, we obtain information about the characteristic strength or strength limit of the material. However, these data are strictly engineering in nature and allow us to determine the strength class of the material or serve as a control tool in the case of quality control of materials used in construction. In order to obtain results of greater scientific value, it is necessary to check the deformation of the tested object under the influence of induced stresses. Information about the deformation of the test object allows the determination of scientifically significant parameters such as stiffness (in various directions), Poisson’s ratio or other material constants. These data are particularly important when modelling the behavior of a structure, or to optimize the composition of an innovative material whose parameters may be difficult to predict. This is particularly true for composite materials. Determining the compressive or tensile strength of a material in uniaxial tension is a relatively straightforward measurement, especially with modern testing machines. It essentially involves reading the pressure required to move the plunger of the testing machine as it compresses the specimen. Historically, in older methods, the compressive or tensile force were measured by checking the deformation of a “calibration ring”.

Reading the deformation of the tested object is already a much more complicated activity due to the different characteristics of the deformations occurring in the object. Deformations of the tested object may be considered as global or local. In the case of global deformations, we consider the response of the material as a whole to the applied loads. We can then measure the change in height or width of the specimen under the influence of applied stresses. This measurement is generally taken as the displacement of the piston compressing the specimen. In the case of local measurements, we focus our attention on a specific part of the test object called the measurement base. Strain gauges that are glued to the surface of the test object are used to make local measurements. They measure the deformation of the tested object on the basis of the change in its resistance caused by shape change. It should be noted that global and local measurements are not identical, and the values obtained do not match due to the influence of the components of the strain distribution.

Modern methods of strain measurement include measurements using digital image correlation (DIC). These measurements use time-lapse video of the test. On the basis of the obtained video recording, software is used to analyze the displacement of points placed on the surface of the tested object. This allows the characteristics of the strain distribution on the surface of the specimen to be determined globally as well as locally.

## 2. Methods of Measuring Deformation of Tested Objects under Uniaxial Stress

**Figure 1:**
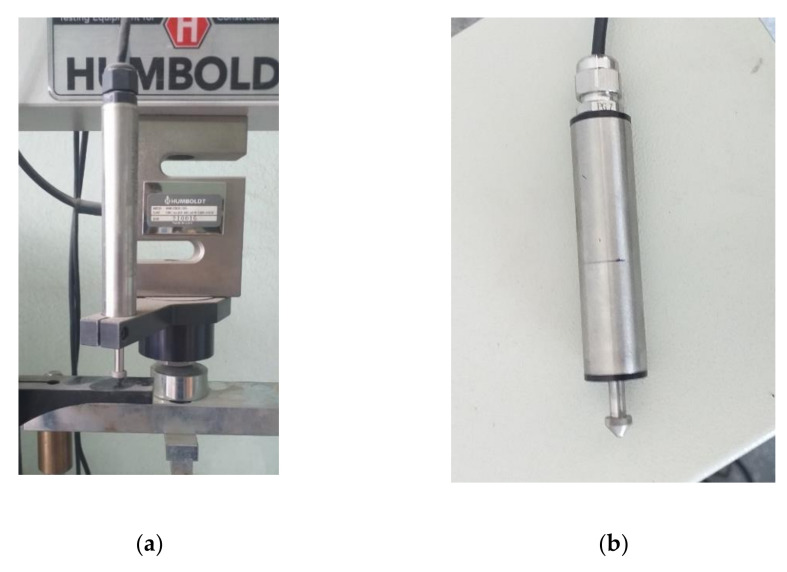
(**a**) Piston displacement measuring adapter; (**b**) linear strain gauge (photo: K. Walotek).

The simplest way to measure the global deformation of a specimen in a strength test is to measure the length of the piston displacement that exerts a force on the specimen (Figure 1 shows a measurement adapter with linear strain gauge). However, the accuracy of this measurement is relatively low. It only allows for a global measurement of deformation and is subject to error due to the possibility of movement of usually jointly mounted, clamping elements, clearance play between the sample and contact elements, and deformation of the clamping elements themselves. Thus, it can be assumed that based on the measurement of the piston displacement we obtain the deformation of the specimen, which can be described by the formula [38]:

(1) Δh=Δu−ΔH

where:

Δ*h*—sample shortening
Δ*u*—piston displacement
Δ*H*—sum of the changes in length of the clamping elements and in clearance.

Because of the aforementioned inaccuracies, this type of deformation measurement is useful only for simple measurements of preferably homogeneous materials, where global influences are of greatest importance.

## 3. Deformation Measurement Using Digital Image Correlation DIC System with the Example of ARAMIS 3D GOM Measurement System

### 3.1. Structure of ARAMIS 3D GOM Measurement System

The ARAMIS 3D system consists of two high-resolution digital cameras, a beamformer (HUB), a blue light source and a counting unit (laptop). The beams (HUB) allow for communication between the cameras and the computing unit and ensure that the cameras have the correct focal length. The blue light source illuminates the tested object, improving measurement accuracy. The computational unit is equipped with appropriate software to perform analysis and calculations based on the measurement data (from the testing press) and recorded video footage.

**Figure 2:**
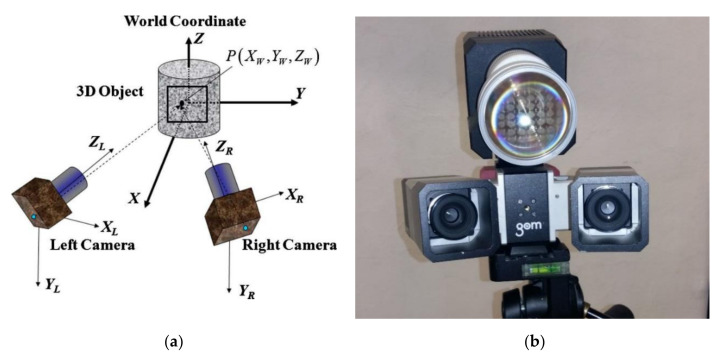
(**a**) Diagram of image acquisition by ARAMIS 3D system [40]; (**b**) ARAMIS 3D measurement system (photo: K. Walotek).

By combining two cameras aimed at a common focal length (Figure 2), the measuring system records the image as a three-dimensional image. Thus, after calibration of the device, automatic measurements can be performed and the ARAMIS system is able to independently read the actual size of the objects tested. The measurement area covered by the cameras depends on the spacing and the angle of the measuring beam (HUB). As the size of the tested object increases, it is necessary to use larger and larger measuring beams. Table 1 provides an overview of the available measuring beams and their testing areas.

### 3.2. Operation Principle of ARAMIS 3D Measurement System

**Figure 3:**
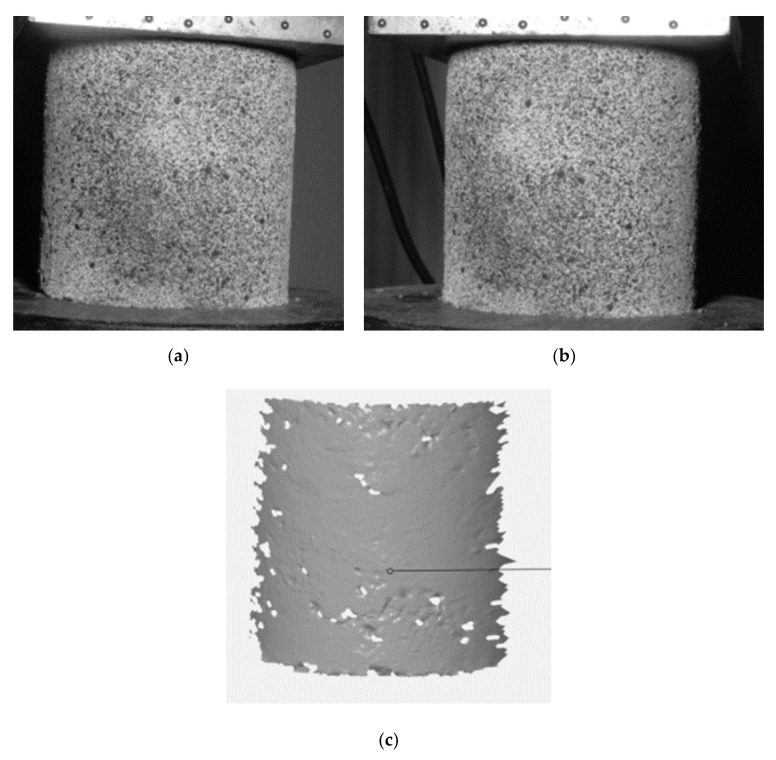
(**a**) Left camera image; (**b**) Right camera image; (**c**) 3D model built from camera images (photo: K. Walotek).

The ARAMIS 3D measurement system takes a series of time-lapse photographs of the object under investigation. On the basis of images taken with cameras of a common focal length, the software tracks and identifies points on the basis of grey scale. The points are identified in each of the consecutive images and their positions are assigned with subpixel accuracy. Then, based on triangulation, the position of each point in the three-dimensional space is calculated by superimposing the images from both cameras. In this way, a three-dimensional model of the surface of the object under study can be built (Figure 3). On the basis of the three-dimensional model of the surface of the tested object, with the help of software we are able to determine the displacements of the points in the three-dimensional coordinate system and their velocities and accelerations caused by the load. In addition, the software can control the deformation of the surface of the test object by creating a map of major or minor deformations.

### 3.3. Surface Preparation of Test Specimens

**Figure 4:**
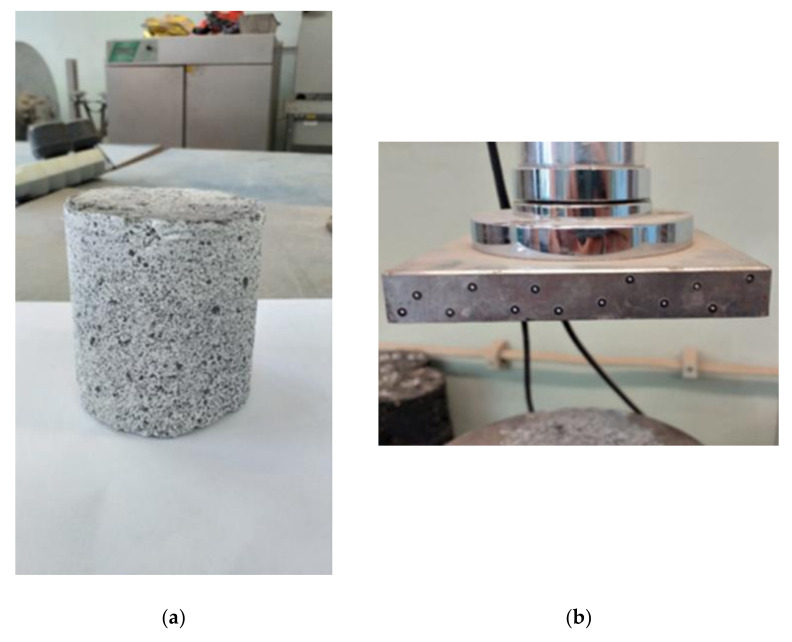
(**a**) Surface of specimen prepared for testing; (**b**) Measurement points on the compression apparatus for cylindrical specimens (photo: K. Walotek).

As previously mentioned, the ARAMIS 3D measurement system determines the position of points on the sample surface on the basis of a gray-scale reading of the surface. To facilitate this, the surface of the specimen must be prepared in an appropriate way. Figure 4a shows a sample prepared for testing. The surface of the specimen should be pre-treated with a white matte paint in order to provide a contrasting and anti-reflective background. Next, a gradient in the form of black spots is applied to the surface of the sample. The spots should be evenly distributed over the sample surface in a 50/50 ratio to the white background. The size and density of the spots depends on the size of the measurement area. In the case of deformation measurements of small objects, the prepared gradient should consist of very fine and densely distributed speckles, while the surface of large objects should be covered with larger speckles with higher dilution.

When measuring the deformation or displacement of fixed components, such as press or pistons adapters, they should be marked with special measurement points such as those shown in Figure 4b. As with the spotted gradient, the size of the measurement points used on the surface of the specimens will depend on the size of the object under test.

## 4. Methodology and Research Object

This paper presents the potential for of using the ARAMIS 3D measurement system to study the effect of the addition of shredded rubber waste on the deformability of binder-bonded specimens.

Samples were prepared for the tests according to Table 2. The samples were compacted in cylindrical 80 × 80 mm molds in two layers with 15 impacts per layer with a lightweight rammer. The samples were compacted at optimum moisture content as determined by the second Proctor method. The testing was done after 28 days of sample maturation. During the maturation, the samples were kept under wet air conditions for 14 days and 14 days under full immersion in water. The paper presents the results of the tests for one sample from the group of five performed for each recipe, G0 and G10.

**Figure 5:**
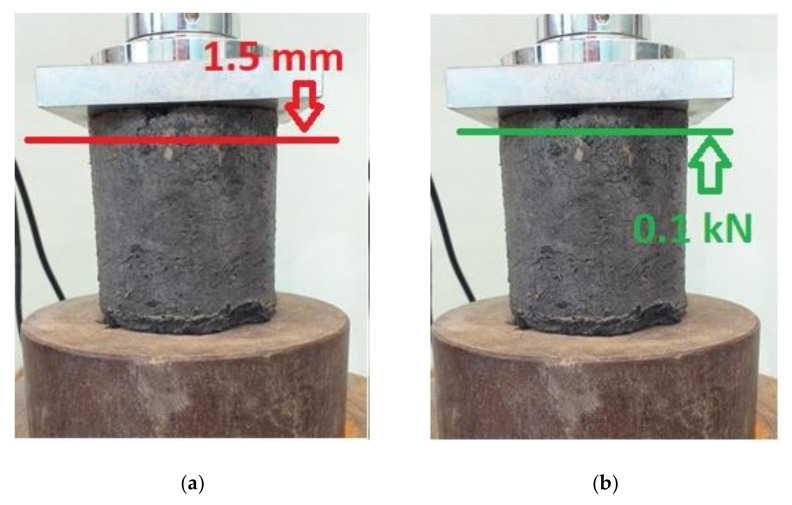
Diagram of (**a**) the loading and (**b**) the unloading cycle of the tested specimens (photo: K. Walotek).

The cyclic loading test was conducted under the conditions shown in Figure 5. Each loading cycle consisted of forcing a global displacement of 1.5 mm on the specimen and then unloading it until a force of 0.1 kN was achieved. Each specimen was preloaded with a force of 2.0 kN to partially eliminate unevenness of the specimen’s contact surface and slack in the testing machine attachments. Each specimen was loaded and unloaded until failure or 20 test cycles were achieved. The loading and unloading were done at a piston speed of 6 mm/min.

## 5. Research Findings and Analysis

### 5.1. Interface Elements and Basic Data Reading Elements

**Figure 6:**
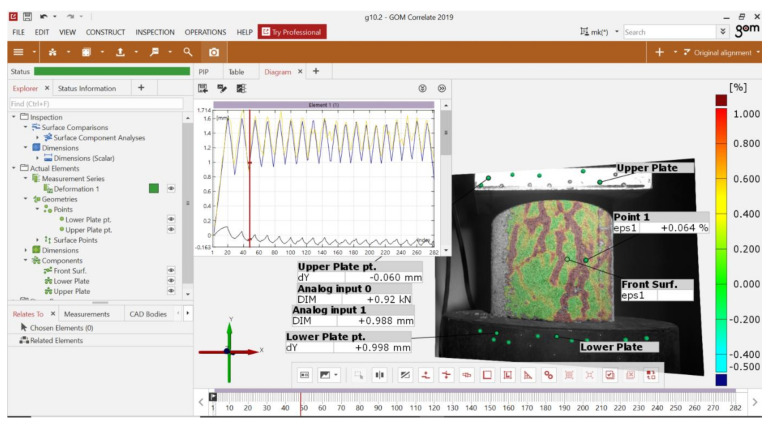
View of ARAMIS 3D measurement system interface.

Figure 6 shows a view of the interface of the ARAMIS 3D measurement system in GOM CORRELATE 2019 software. At the beginning of the test, the fixed elements that are repeated when the test is performed were marked on each successive specimen. The labelled fixed elements are:

- The lower plate the causes displacements on the specimen is labelled as Lower plate. A point component (obtained by using the measurement points of Figure 4b) was marked on this element, based on which the global deformations forced on the specimen were measured.
- The upper plate that imparts resistance to the test specimen is designated Upper plate. On this element, a point component was applied to measure the amount of backlash, which was reset at each loading of the specimen.
- The surface component was obtained from a gradient scan of the sample surface. This component is a 3D model of the surface of the test specimen (Figure 3c). The surface component is used to inspect the major strains occurring on the specimen surface according to the legend shown on the left side of the interface.
- Analog input 0 is the compression force reading from the testing press to which the ARAMIS 3D system is coupled.
- Analog input 1 is the displacement reading of the lower plate of the testing machine; this is a measurement of a value, which is partially identical to the displacement value read from the Lower plate component.

The GOM CORRELATE 2019 interface is standard for the Microsoft Windows environment. The most important elements of the interface are:

- Tree of performed measurements and elements marked on the test image (left side of the interface);
- Sample view and 3D model of the sample surface with selected elements (center of interface);
- Timeline, located at the very bottom of the interface. This marks the individual frames of the recorded test;
- The diagram above the sample preview that allows constant control of the measured parameters in each successive frame of the test recording.

The most common problem when connecting, e.g., testing machines with other measuring devices is the “noise” of the obtained signal. This problem can be solved by using electronic signal amplifiers or filters. However, this is not always possible due to technical specifications of the equipment. In the case of the presented test results, the data obtained from the analog input 0 and 1 sources were noisy due to the differences in the output voltage of the testing machine and the ARAMIS 3D receiver. This can be observed in Figure 6 when comparing the displacement values read from analog input 1 and Lower plate point 1. These measurements should give comparable values, but in the case of analog input 1 there are noticeable deviations from the displacement forcing trend. In the GOM CORRELATE 2019 software there is an option to apply filters to the acquired data to negate the effects of signal noise or poorly executed sample surface painting, which can also affect the results. The filters used in the software are divided into 2 groups:

- Surface filter—is a filter that compensates for the effects of improperly painted samples on the results. This filter compares measurements of neighboring points of a 3D model of sample surface, and rejects results that differ significantly from each other. The numerical value used for the filter can be used to determine the number of adjacent points when verifying the measurement.
- Temporary filter—this filter verifies the scatter of measurement results between the same measurement points in consecutive frames. The numerical value used for this filter defines the number of neighboring frames to be taken into account while verifying the measurement.

In addition to the surface filter and a temporal filter, the type of filter can be selected within each of these categories. The type of filter corresponds to the mathematical method on the basis of which the results will be verified. The software provides the application of the following mathematical methods:

- Average filter—averages the values obtained in adjacent points of the plot or adjacent frames of the recording;
- Median filter—determines the median of the values obtained at adjacent points of the plot or frames of the recording;
- Binomial filter—based on the binomial function, verifies measurements of adjacent points of the surface or frames of the recording;
- Spline smoothing—this is a method for graphically smoothing the results obtained without interfering with their numerical values.

**Figure 7:**
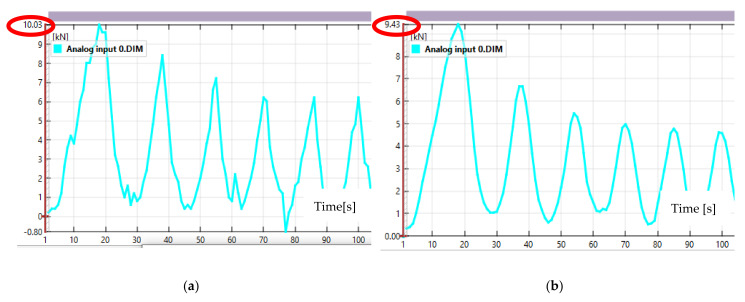
Force values read from analog input 0 source: (**a**) without applying filters; (**b**) after applying a temporary filter with a value of “2”.

**Figure 8:**
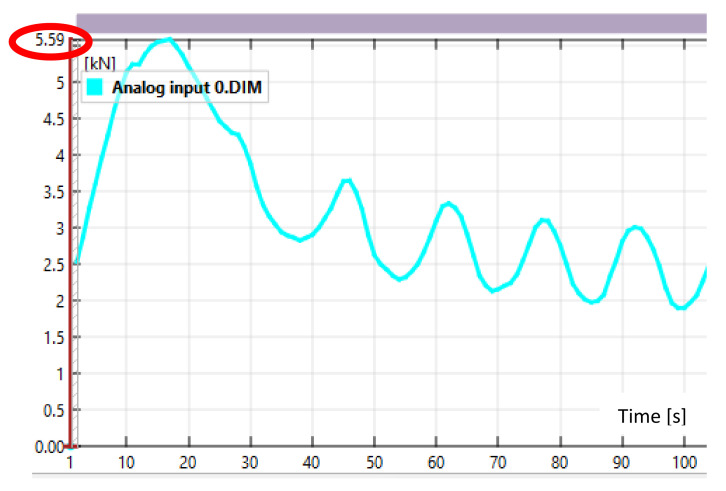
Force values read from source analog input 0 when using the wrong filter numerical value.

Figure 7 shows an example of using temporary filters to suppress “noise” from the analog input 0 source, which reads the compression force from the test press. In Figure 7a, the force reading is noisy, with significant spikes in the readings. This is especially noticeable in the maximum and minimum areas. Figure 7b shows the same force measurement, but after applying a temporal average filter based on two adjacent test frames. As can be seen, after applying the filter the results show a much smoother trend in the compressive force change without major jumps or breaks. However, as a result of the filter application, the maximum force at each of the peak values has decreased. Therefore, the application of particular filters and the reliability of the results obtained with them depends mainly on the experience of the software operator. If the wrong filter is selected or the numerical value attached to it is wrong, significantly deviated results will be obtained, as in Figure 8 where the mean filter was applied again to filter the results shown, but with a frame value of 10.

As can be seen, after applying the erroneous value of the controlled adjacent frames of the recording, the readings decreased significantly and the trend of the compressive force change is very different to the baseline reading.

### 5.2. Measuring Capabilities of ARAMIS 3D

The measurement capabilities of the ARAMIS 3D operating system are very broad. In this article, the authors only focus on the most important of them. The most important and basic measurement offered by the ARAMIS 3D system is the measurement of displacements occurring on the surface of the sample. The basic parameters that can be measured on the surface of the tested object are presented below. These include:

- Measurement of displacements oriented in the X/Y/Z directions of the assumed coordinate system;
- Mises’ balanced strains;
- Thickness reduction;
- Distortion in vertical and horizontal planes, without depth control;
- Major and minor strains.

The most commonly used measurement is the major strain measurement, which allows the most stressed areas of the surface of the test piece to be determined.

**Figure 9:**
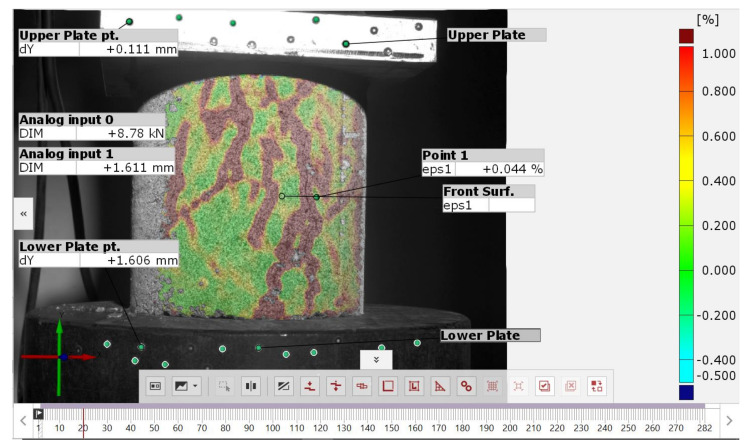
Image of the major strains of the specimen surface at the peak load of the first test cycle.

Figure 9 shows a view of the major strain map on the specimen surface at the peak load of the first test cycle. This image allows us to determine where scratches have developed in the specimen that may lead to a reduction in load capacity or failure of the test piece. Based on the scratch location, it is possible to perform a spot check for strain development at the crack propagation location or determine at what deformation or stress threshold the failure of the test object occurs. This makes it possible to collect information about the material after the test, and to precisely determine the values of the tested parameters in the areas of greatest importance for the operation of the tested object.

**Figure 10:**
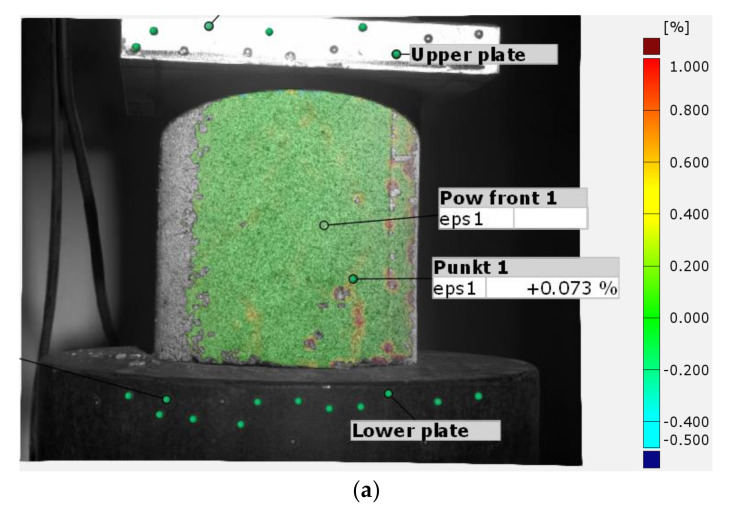

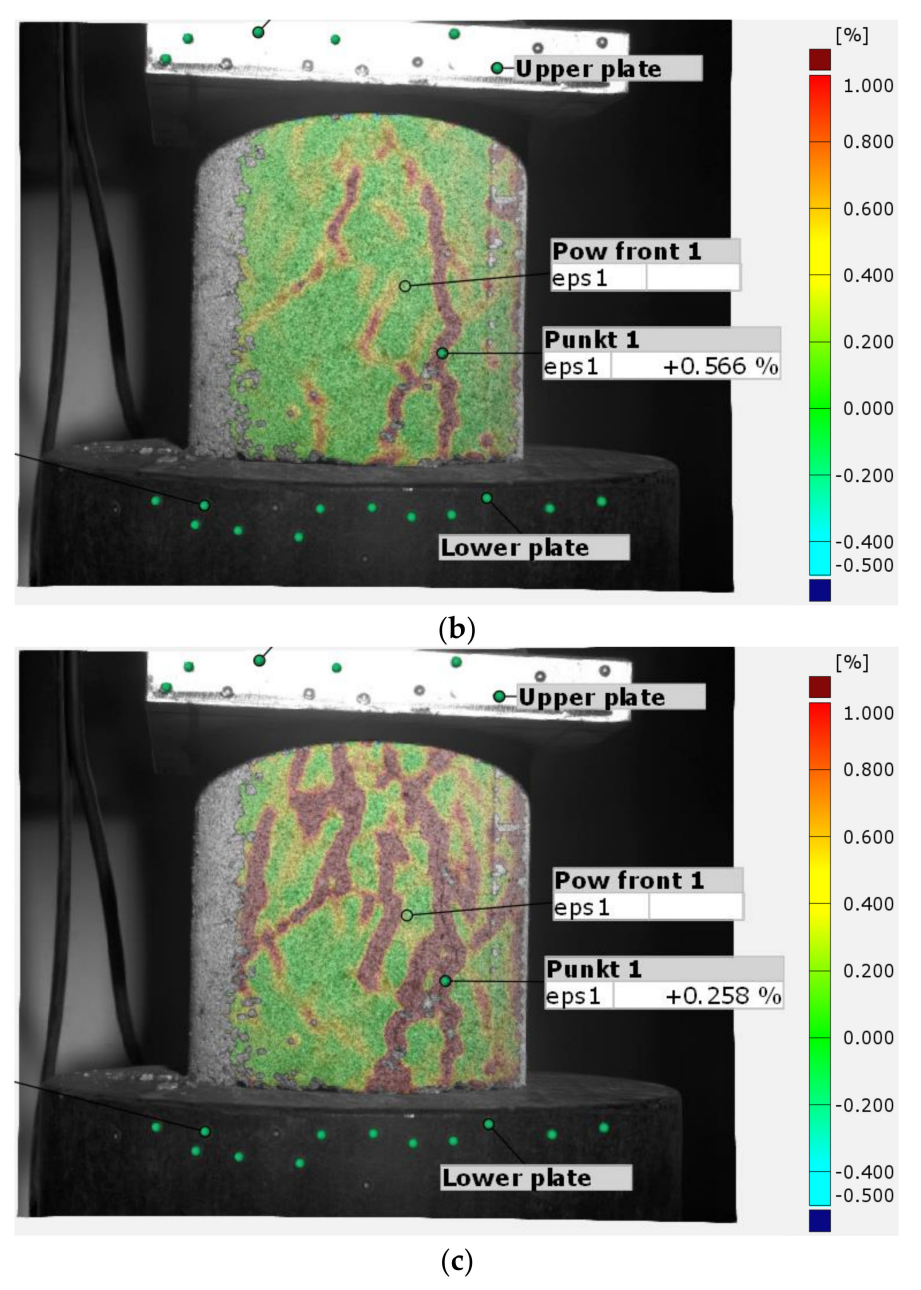
Inspection of major strain in a scratch during its propagation in: (**a**) The beginning of the load; (**b**) before peak load; (**c**) after the peak load.

Point strain inspection (Figure 10), performed on the basis of the strain distribution on the specimen surface, allows for accurate characterization of the influence of local phenomena on the work of the entire test specimen. This is particularly important in the case of composite materials, where local contact issues between individual components of the composite will determine its global work.

**Figure 11:**
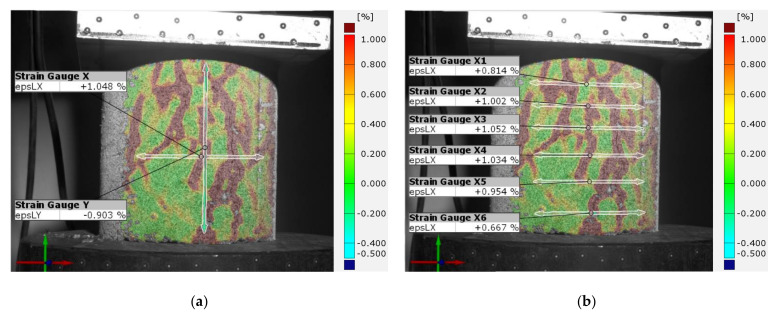
Example of placing virtual strain gauges on the sample surface: (**a**) measurement in perpendicular planes; (**b**) measurement in parallel planes—measurement of lateral strains in planes arranged at different heights of the sample.

**Figure 12:**
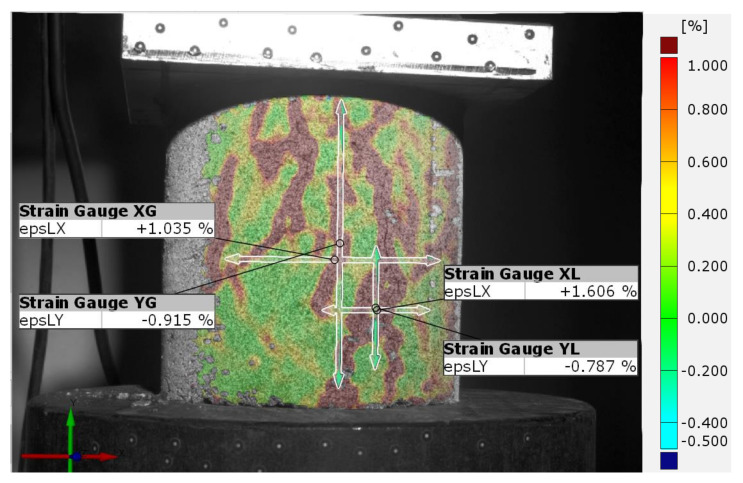
Example of using strain gauges to measure deformation at the specimen surface and in the emerging scratch.

In addition to the control of deformations occurring on the entire surface of the tested specimen and point control of local phenomena, the ARAMIS 3D measurement system software allows virtual strain gauges to be placed on the surface of the specimen. Strain gauges are a basic tool for checking local deformations of the object in planes consistent with the axis of load application and transverse to the axis of load application. However, unlike classical glued strain gauges, in this case we can again decide on its location and the size of its measuring base, based on observation of the specimen’s behavior during testing. Figure 11a shows an example of the location of virtual strain gauges on the sample surface. The strain gauges are located at the center of gravity of the specimen in a longitudinal and transverse direction to the load axis. The data obtained helps to determine important material constants, such as Poisson’s ratio or stiffness in different directions depending on the axis of load application. Classical strain gauges can provide similar data, but the advantage of the DIC system is the ability to apply any number of transverse strain gauges equally spaced along the height of the specimen (Figure 11b). This allows the transverse strain distribution to be determined as a function of the height of the strain gauge location. In another case, it is also possible to overlay strain gauges with small measurement bases in areas of local phenomena that may determine the global behavior of the test object (Figure 12).

### 5.3. Example of ARAMIS 3D Measurement Capabilities

**Figure 13:**
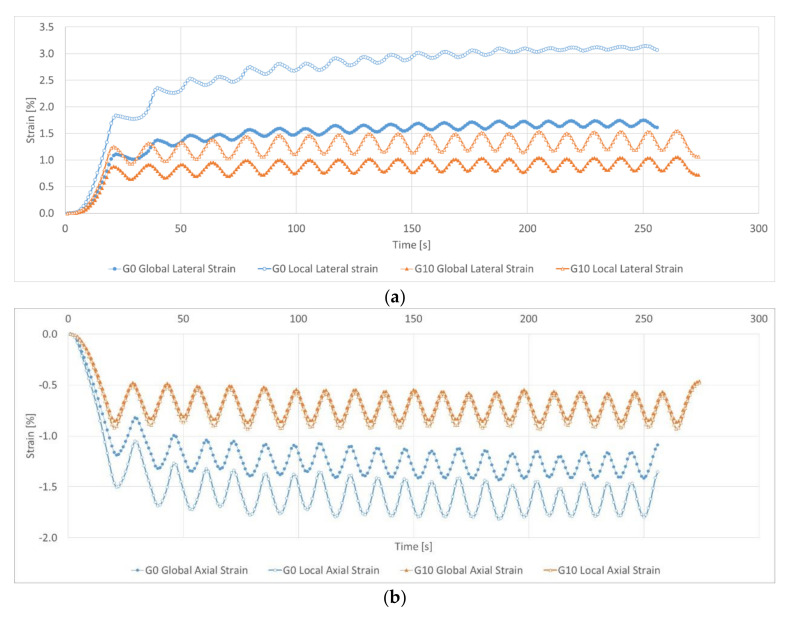
Time dependence of global and local strains (cyclic loading of samples): (**a**) lateral strains of G0 and G10 samples; (**b**) axial strains of G0 and G10 samples.

**Figure 14:**
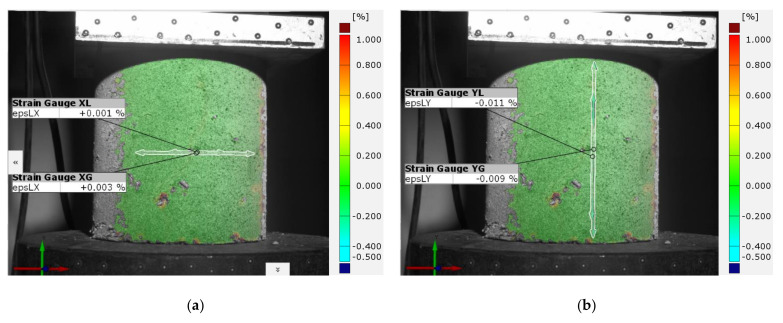
The arrangement of global and local strain gauges (**a**) for lateral measurements, (**b**) for axial measurements.

Figure 13 shows the values of lateral strains (a) and axial strains (b) during the cyclic load test. The strains were measured with virtual global and local strain gauges located on the surface of sample. The global strain gauges were 73 mm long in the longitudinal direction and 53 mm in the transverse direction, and the local strain gauges were 53 mm long and 25 mm transverse. Global and local strain gauges were placed in the same planes and axes on the sample surface (Figure 14).

In the case of the diagram in Figure 13a, the values of the lateral strains of the tested samples were analyzed. The sample made according to the formula G0 has a global lateral strain of about 1.10% at the first load, then the value of the lateral strain systematically increases up to the 11th load cycle, where it is about 1.75% under load and 1.55% under unloading. On the basis of these values, it can be concluded that the tested sample works elastically in the strain range of 0.20%. In the case of the local lateral strains in the tested sample, the measured values do not show any visible stabilization. They increase with each load and unload cycle of the sample. Additionally, the values of the lateral strains read from the local strain gauge are much higher, ranging from approximately 1.80% in the first load cycle to 3.20% in the 20th cycle. The amplitude of the lateral strains under loading and unloading in the last test cycles (cycle 14–20) also noticeably decreases. This change proves the gradual limitation of the scope of the elastic work of the material through the propagation of the resulting scratches.

In the first test cycle, the sample made according to the G10 formula obtains global lateral strains equal to approximately 0.95% with loading and 0.65% with unloading. These values were reached in the fourth cycle. With each subsequent test cycle, these values increased slightly up to 1.10% under load and 0.80% under unloading in the last test cycles. At the beginning and the end of the test, the global deformation amplitude remains practically unchanged at the level of about 0.30%. This proves that the range (number of cycles) of elastic work has increased with a gradual, very slow deepening of the range of plastic deformations (cycle 5–20). The measurement of lateral strains was performed with a local strain gauge, and similar to the G0 sample, presents higher values than the global strain gauge. However, the difference is much smaller. In the first load cycle, the local strain gauge showed the values of lateral strains equal to approximately 1.15% with loading and 0.90% with unloading. These values increased again with each test cycle until they reached a value of approximately 1.60% under load and 1.20% under unloading for the last test cycles. In the case of the local strain gauge, the initial elastic work range was about 0.35% and increased to about 0.4% in the last test cycles.

We used absolute values when analyzing the results of the axial strains (Figure 13b); the strain results have negative values due to the compression of the sample in the longitudinal direction. For the sample without the addition of rubber fines (G0), the global axial strains reached about 1.40% with loading and about 1.09% with unloading in the fifth cycle. Further, the values of axial strains under load stabilize, while when unloading, we observe a further change in readings until a value of about 1.17% was reached. This means a change in the range of elastic work from about 0.31% in the fifth cycle to about 0.23% in the last cycle. Local strain gives higher readings, ranging from about 1.79% with load and about 1.37% with unload on the fifth cycle. As in the case of global strains under load, this value stabilizes in subsequent cycles, while strains under unloading continue to propagate, reaching about 1.47% in the last cycles. Again, we observed a change in the range of elastic work from about 0.42% in the fifth cycle to about 0.32% in the last cycles.

The results of axial strains for the sample with rubber fines (G10) are characterized by the fact that firstly, we obtained lower values of axial strains, secondly, global and local strains have almost identical values, and thirdly, an almost constant range of elastic work was observed from the first cycle to the last one. Axial strains were already stabilized in the first load cycle, reaching values of about 0.85% for the global measurement and about 0.92% for the local measurement. Measurements during unloading in the first cycle reached a value of about 0.50% for the global strain gauge and about 0.51% for the local strain gauge. The strain value stabilization takes place in the sixth cycle, reaching values of about 0.56% for global strains and about 0.60% for local strain. On this basis, it can be concluded that in the first load cycles, the plastic deformation is exhausted and in subsequent cycles the work of the sample is only subject to elastic work (cycles 6–20). This applies to both local and global strains. The range of elastic work after stabilization of elastic deformations (from the 6th cycle) for global measurements was 0.29%, while for local measurements it was 0.32%.

**Figure 15:**
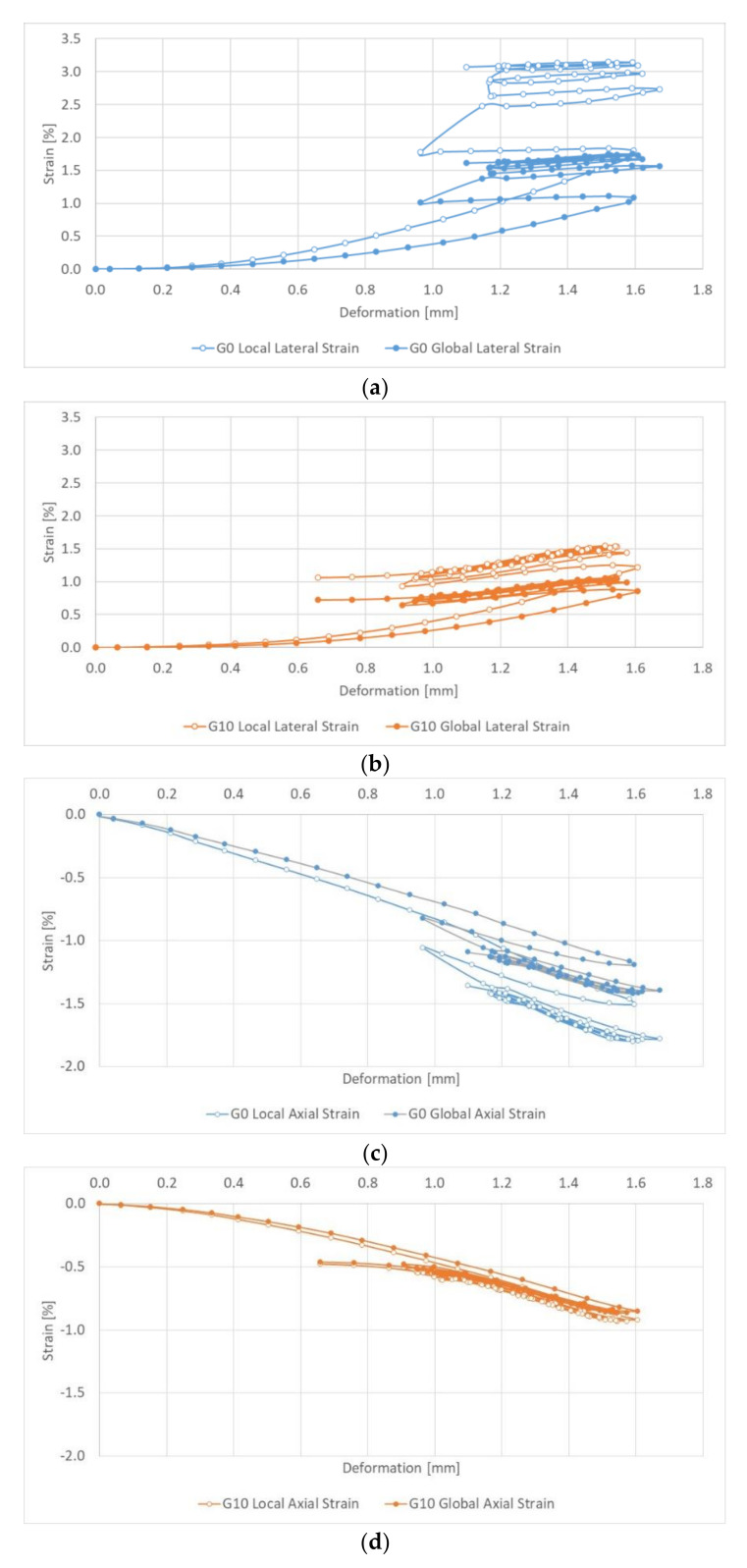
Dependence of global and local strains on axial samples deformation: (**a**) lateral strains G0; (**b**) lateral strains G10; (**c**) axial strains G0; (**d**) axial strains G10.

Thanks to the capabilities of the ARAMIS 3D system, the graphs in Figure 15 show the dependence of horizontal and vertical strains on the deformation of the sample. The results are presented in the form of deformation hysteresis. For clarity, only the results for selected load and unload cycles are presented in the diagrams; these include cycles 1, 5, 10, 15 and 20. When the dependencies are presented in this way, it is possible to find even clearer differences in the way the samples work, especially for the G0 series but also for the G10 series, depending on the method of measuring.

The method for processing the results, which is based on the analysis of recorded and appropriately prepared images, allows us to perform a back-analysis of the results obtained in this way. This makes it possible to measure and obtain results anywhere in the measurement area, and also at any moment (time (s)) during the recorded test. In the presented example, it was possible to change/supplement the results of global measurements with local measurements. Further, it was possible to analyze the behavior of axial and lateral strains in time, i.e., in successive cycles of loading and unloading. In classic methods based on physical sensors (strain gauges), the results are obtained only in the place where the sensor is mounted, without the possibility of supplementing the measurement data later.

## 6. Summary

On the basis of the presented research, by using DIC technologies it was possible to establish the influence of fragmented rubber waste on the deformability of the tested composite. The shredded rubber waste reduces the stiffness of the tested composite and allows a larger volume of the sample to be pulled together while it is compressed. In addition, the authors were able to determine the Poisson’s ratio for both tested samples (Table 3). The Poisson’s ratio was determined as 30% of the maximum force applied to the sample in accordance with [41]. The G0 sample obtained a Poisson’s ratio of 0.25, which is the highest limit value obtained by cement concrete. In the case of the G10 sample, the 10% addition of shredded rubber waste increased the value of the Poisson’s ratio to 0.28, which proves its influence on the specified parameter.

Measurement systems based on DIC technology, such as ARAMIS 3D, allow us to make very accurate measurements of displacements occurring on the surface of the tested objects. The measurements produce results of high quality, even in the case of testing elements with complex surface structures, due to the automatic depth measurement and construction of 3D model of the seen surface. The DIC system incorporates all of the measurement methods used in materials testing. They allow us to perform both global and local deformation measurements, where in case of local measurements it is possible to precisely define the tested area, point or type of measurement. However, the most important aspect of these DIC systems is the ability to decide and make additional strain measurements after the specimen has already been subjected to impact damage. This allows us to get a general idea of the deformation that is occurring on the surface of the specimen, and then determine the most important areas or parameters for the overall behavior of the specimen. This approach also allows multiple series and types of measurements to be performed on a single specimen, reducing the amount of laboratory work required to prepare the specimen for testing. The characteristics of DIC systems means they are very often used in aerospace and automotive industries, where precise measurements of deformations of tested objects in fatigue or failure conditions are important for the safety of users of civil and air transport [42]. The use of DIC systems should be increasingly used in the coming years in the testing of construction materials, where due to the freedom of measurement they could reduce the number of required test objects, which generally require a long time to mature before testing. In addition, such systems are very well suited for testing composite materials, which are becoming increasingly common in the construction industry due to, among other things, the use of waste materials as raw materials for production.

## Figures and Tables

**Table 1:** Summary of ARAMIS 3D measurement beams [39].

Measuring Beam	Measurement Focal Distance (mm)	Measuring Area (mm)
150 mm	350	30 × 20 × 10
	350	60 × 50 × 30
	350	100 × 80 × 50
	350	150 × 120 × 90
300 mm	700	150 × 120 × 90
	700	215 × 180 × 150
	700	330 × 270 × 200
	700	600 × 530 × 400
600 mm	1400	680 × 560 × 560
	1400	1250 × 1100 × 1100
1200 mm	2700	1350 × 1100 × 1100
	2700	2500 × 2150 × 2150
1600 mm	4500	5000 × 4400 × 4400

**Table 2:** The recipes for the mixtures used for the research.

Recipe	Unburnt Coal Mining Slate 0/16 mm (%)	Shredded Rubber Waste 0/2 mm (%)	Silica Fly Ash (%)	CEM I 42.5 R (%)	Water Content * (%)
G0	90	0	5	5	11.2
G10	80	10	5	5	10.1

* amount of the added water in relation to the dry weight of the other ingredients.

**Table 3:** Poisson’s ratio values for samples G0 and G10.

	G0	G10
F_30%_ (kN)	3.816	2.031
Global axial strain in F_30%_ (%)	−0.275	−0.101
Global lateral strain in F_30%_ (%)	0.068	0.028
Poisson’s ratio ν (-)	0.25	0.28

## Data Availability

Data sharing not applicable.
